# A Rare Case of Endometrial Cancer Metastatic to the Sigmoid Colon and Small Bowel

**DOI:** 10.1155/2017/9382486

**Published:** 2017-10-25

**Authors:** Jeffrey A. Hubers, Anurag Soni

**Affiliations:** Division of Gastroenterology & Hepatology, University of Wisconsin School of Medicine & Public Health, Madison, WI, USA

## Abstract

Metastatic endometrial cancer to the small bowel or colon has been described but is quite rare. We present a case of metastatic endometrial cancer with synchronous metastases to the colon and jejunum identified three years after surgical treatment of early stage endometrial cancer.

## 1. Introduction

Blood per rectum and iron deficiency anemia are two common indications for performing a diagnostic colonoscopy to evaluate for malignant tumors of the colon. Primary colonic adenocarcinomas account for the majority of malignant tumors identified at colonoscopy. Less commonly, metastasis from other primary cancers, including breast, ovary, prostate, lung, and stomach, can present as a colonic tumor [1]. Here, we present a case of metastatic endometrial cancer to the bowel in a patient with a remote history of endometrial cancer.

## 2. Case Report

A 75-year-old female with a history of endometrial cancer presented to gastroenterology clinic with one month of blood per rectum. She also described bilateral lower abdominal pain but no weight loss, diarrhea, or constipation. CT of the abdomen and pelvis with PO and IV contrast completed one month prior showed no findings of recurrent or metastatic disease. Colonoscopy eight years before presentation was notable for diverticulosis without polyps.

The patient had a past medical history of FIGO (International Federation of Gynecology and Obstetrics) grade 1 stage IB endometrioid adenocarcinoma diagnosed three years prior and treated with total laparoscopic hysterectomy and bilateral salpingooopherectomy. Pelvic and periaortic lymph node dissection showed no malignancy in 28 of 28 sampled lymph nodes. Postoperatively, four fractions of intracavity brachytherapy to the vaginal cuff were completed, for a total radiation dose of 22 Gy. The patient also had a history of diverticulitis seven years prior treated with partial sigmoid colectomy, type II diabetes, and obesity. On physical exam, there was abdominal tenderness with moderate palpation of the left mid-quadrant of the abdomen. The rest of the exam was unremarkable. Labs showed a hemoglobin level of 10.3 g/dL, down from 12.9 g/dL one year prior. An EGD and colonoscopy were ordered.

**Figure 1:**
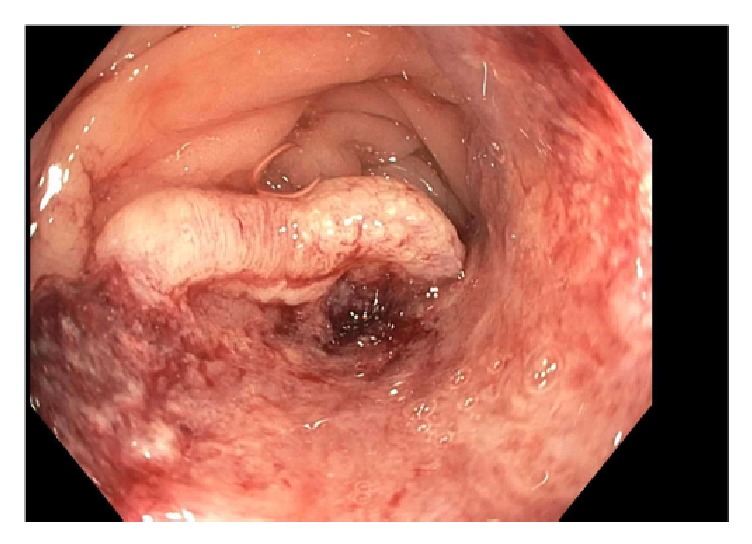
Large, ulcerated mass in the sigmoid colon seen during colonoscopy.

**Figure 2:**
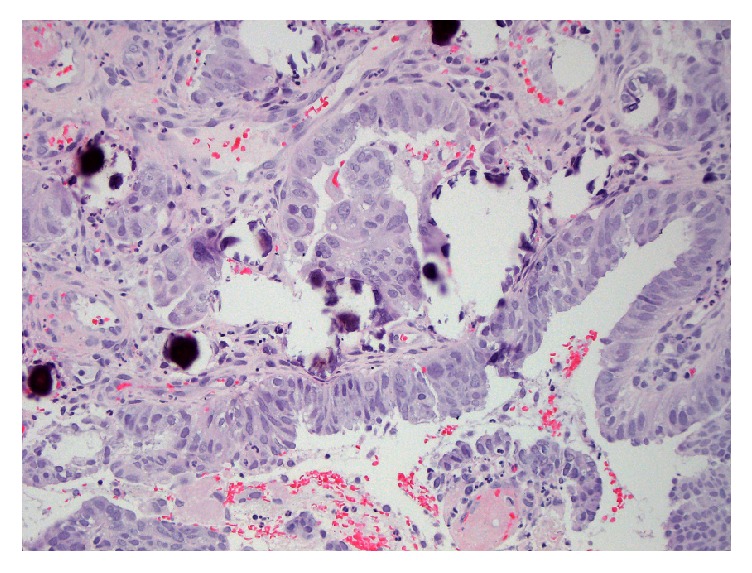
Hematoxylin and eosin staining shows fragments of large atypical cells with focal gland formation consistent with an adenocarcinoma.

**Figure 3:**
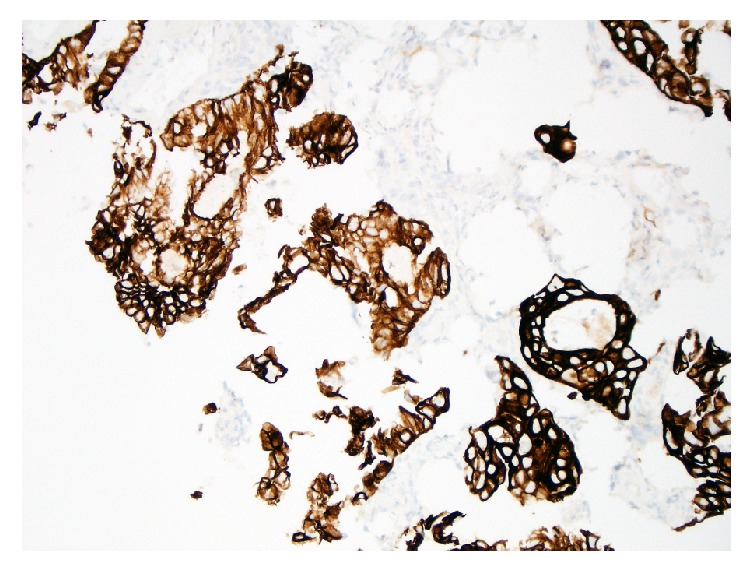
Cytokeratin 7 (CK7) typically stains positive (dark brown) in carcinomas of epithelial origin including endometrial cancer.

The upper endoscopy was unremarkable. Colonoscopy showed a 2 cm, nonobstructing, ulcerated mass in the sigmoid colon (Figure 1). Biopsies of the sigmoid mass were consistent with an adenocarcinoma (Figure 2). By immunohistochemistry (IHC), the cells were positive for CK7 (Figure 3), PAX-8, and ER and negative for CDX-2, CK20, and WT-1. Prior slides of the patient's past endometrial adenocarcinoma were reviewed and the overall histology was similar to the colonic biopsies. Based on the IHC and histology, a diagnosis of metastasis from the prior endometrial adenocarcinoma was made.

**Figure 4:**
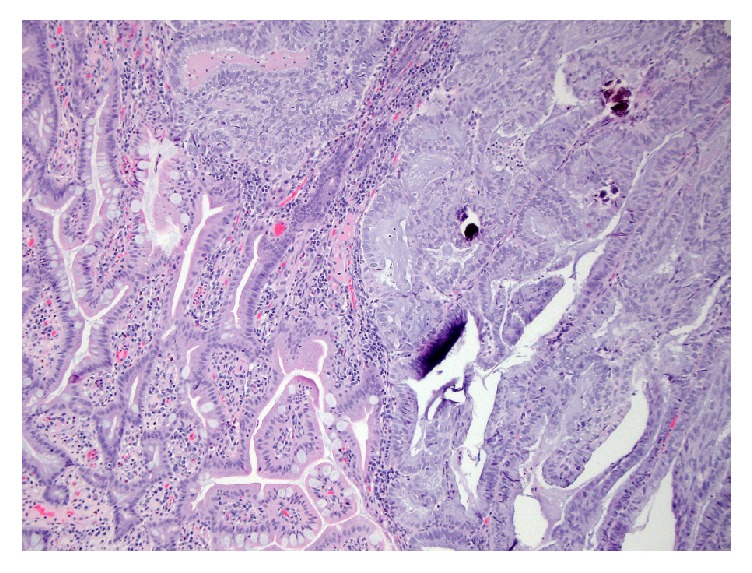
Normal colon mucosa on the left with the margin of tumor on the right containing large, atypical cells consistent with adenocarcinoma.

The patient underwent an exploratory laparotomy. The colorectal surgeons considered a laparoscopic approach but due to the anticipated adhesions from previous abdominal surgeries, a laparotomy was performed. Intraoperatively, a 1.8 × 1.8 cm sigmoid mass was found, along with a 1-2 cm proximal jejunum mass involving the wall of the bowel. A low anterior resection with primary colonic anastomosis was performed, along with a small bowel resection with primary anastomosis. Histology of the surgical specimen confirmed metastatic endometrioid adenocarcinoma in the small bowel and sigmoid tumors (Figure 4). Omental biopsies, in addition to peritoneal fluid washings, were negative for carcinoma.

Postoperatively, oncology providers planned for six cycles of chemotherapy with paclitaxel 175 mg/m^2^ BSA and carboplatin 500 mg, adjusted for creatinine clearance. Only five cycles were able to be completed due to the development of grade 2 neuropathy, refractory to gabapentin treatment. Follow-up CT imaging six months after surgery showed no evidence of recurrent disease.

## 3. Discussion

Endometrial cancer is the fourth most common cancer in women in the United States and ranks sixth in cancer-related deaths among women [2]. Endometrial cancer has many histologic subtypes, with endometrioid adenocarcinoma being the most common. Staging of endometrial cancer is performed surgically and classified according to the FIGO staging classification. FIGO stage I represents cancer that is confined to the body of the uterus. Further subclassification depends on the depth of invasion of the tumor. FIGO stage IB endometrial cancer represents tumor that invades more than 50% of the myometrium [3], as was seen in this patient. Stage I disease is treated with total hysterectomy and bilateral salpingooophorectomy with or without radiation. Several risk factors for recurrence in early stage endometrial cancer have been identified including histologic grade 3, depth of myometrial invasion (>50%), and age > 60 years [4], along with lymphovascular invasion and lower uterine involvement. Guidelines from the National Comprehensive Cancer Network (NCCN) on uterine neoplasms state adjuvant radiation can be considered if two of the above risk factors are present, although current studies have not shown an overall survival benefit with this approach [5]. Since our patient had two risk factors (age > 60 years old and tumor with >50% myometrial invasion), she underwent adjuvant radiation therapy with vaginal brachytherapy. Typical sites of recurrence are locoreginal organs including the vagina and pelvic lymph nodes. Atypical sites include lungs, bones, brain, and visceral organs [6]. Our patient developed synchronous disease recurrence at two atypical sites in the bowel without involvement of the peritoneum or omentum.

Immunohistochemistry (IHC) can help identify the primary site of malignant tumors, especially poorly differentiated carcinomas. The expression of cytokeratins 7 and 20 (CK7 and CK20) can be helpful in the diagnosis of carcinomas of epithelial origin, particularly in discriminating metastatic colon and ovarian carcinoma. Nearly all cases of colorectal carcinoma are CK7− and CK20+, whereas 100% of endometrial cancers were CK7+ and CK20− [7]. Endometrioid carcinomas can also stain positive for ER, PR, and CA 125 [8]. CDX-2 immunostaining is positive in nearly all cases of primary colorectal adenocarcinomas; however, secondary adenocarcinomas that arose outside of the gastrointestinal tract were typically negative for CDX-2 [9], as was observed in this patient.

Endometrial cancer metastasis to the bowel has been described but is rare, with less than ten published cases. Two of these cases involved early stage endometrial cancers (FIGO stage IB) [10, 11]. Endometrial cancer has also been discovered to arise as malignant transformation from colonic endometriosis [12]. These usually arise from the serosa layer of the colon wall. Our patient had no history of endometriosis or findings of endometriosis on surgical pathology. On review of the literature, only one other case described endometrial cancer metastasis to the colon in the absence of endometriosis. That particular patient had a history of FIGO grade 2 stage IB endometrial cancer with a new finding of a sigmoid colon tumor, initially diagnosed and treated as primary colonic adenocarcinoma. However, further analysis of the tumor with IHC led to a diagnosis of metastatic endometrial cancer to the colon [13]. To our knowledge, no previous cases of synchronous metastases to the small and large bowel have been described. While rare, it is important for gastroenterologists, along with pathologists and oncologists, to keep metastatic disease in the differential diagnosis of tumors in the intestine.

## References

[1] Bresalier R. S., Feldman M., Lawrence S., Brandt L. J. (2015). Colorectal Cancer. *Sleisenger and Fordtran's Gastrointestinal and Liver Disease*.

[2] American Cancer Society. Cancer Facts &amp; Figures 2017. Atlanta: American Cancer Society, 2017

[3] FIGO Committee on Gynecologic Oncology (2014). FIGO staging for carcinoma of the vulva, cervix, and corpus uteri. *International Journal of Gynecology & Obstetrics*.

[4] Dunn E. F., Geye H., Platta C. S. (2014). Predictive factors of recurrence following adjuvant vaginal cuff brachytherapy alone for stage i endometrial cancer. *Gynecologic Oncology*.

[5] Koh W.-J., Greer B. E., Abu-Rustum N. R. (2014). Uterine neoplasms, version 1.2014. *JNCCN — Journal of the National Comprehensive Cancer Network*.

[6] Kurra V., Krajewski K. M., Jagannathan J., Giardino A., Berlin S., Ramaiya N. (2013). Typical and atypical metastatic sites of recurrent endometrial carcinoma. *Cancer Imaging*.

[7] Chu P., Wu E., Weiss L. M. (2000). Cytokeratin 7 and Cytokeratin 20 expression in epithelial neoplasms: a survey of 435 cases. *Modern Pathology*.

[8] Mittal K., Soslow R., McCluggage W. G. (2008). Application of immunohistochemistry to gynecologic pathology. *Archives of Pathology & Laboratory Medicine*.

[9] Groisman G. M., Bernheim J., Halpern M., Brazowsky E., Meir A. (2005). Expression of the intestinal marker Cdx2 in secondary adenocarcinomas of the colorectum. *Archives of Pathology & Laboratory Medicine*.

[10] Bosscher J., Barnhill D., O'Connor D., Park R. (1994). Clinical Stage IB Endometrial Adenocarcinoma with an Isolated Small Bowel Metastasis. *Gynecologic Oncology*.

[11] Gallotta V., Nero C., Callari C. (2016). Laparoscopic Management of a Small Bowel Recurrence of Endometrial Cancer. *Journal of Minimally Invasive Gynecology*.

[12] Petersen V. C., Underwood J. C. E., Wells M., Shepherd N. A. (2002). Primary endometrioid adenocarcinoma of the large intestine arising in colorectal endometriosis. *Histopathology*.

[13] Anstadt M. J., Lapetino S. R., Defnet A., Kapur U., Shoup M. (2012). Endometrial adenocarcinoma metastatic to the colon masquerading as a primary colon cancer. *Journal of Gastroenterology and Hepatology Research*.

